# Applications of a Hyperspectral Imaging System Used to Estimate Wheat Grain Protein: A Review

**DOI:** 10.3389/fpls.2022.837200

**Published:** 2022-04-08

**Authors:** Junjie Ma, Bangyou Zheng, Yong He

**Affiliations:** ^1^Institute of Environment and Sustainable Development in Agriculture, Chinese Academy of Agricultural Sciences, Beijing, China; ^2^CSIRO Agriculture and Food, Queensland Bioscience Precinct, St. Lucia, QLD, Australia

**Keywords:** hyperspectral imaging, wheat, grain protein, vegetation index, machine learning

## Abstract

Recent research advances in wheat have focused not only on increasing grain yields, but also on establishing higher grain quality. Wheat quality is primarily determined by the grain protein content (GPC) and composition, and both of these are affected by nitrogen (N) levels in the plant as it develops during the growing season. Hyperspectral remote sensing is gradually becoming recognized as an economical alternative to traditional destructive field sampling methods and laboratory testing as a means of determining the N status within wheat. Currently, hyperspectral vegetation indices (VIs) and linear nonparametric regression are the primary tools for monitoring the N status of wheat. Machine learning algorithms have been increasingly applied to model the nonlinear relationship between spectral data and wheat N status. This study is a comprehensive review of available N-related hyperspectral VIs and aims to inform the selection of VIs under field conditions. The combination of feature mining and machine learning algorithms is discussed as an application of hyperspectral imaging systems. We discuss the major challenges and future directions for evaluating and assessing wheat N status. Finally, we suggest that the underlying mechanism of protein formation in wheat grains as determined by using hyperspectral imaging systems needs to be further investigated. This overview provides theoretical and technical support to promote applications of hyperspectral imaging systems in wheat N status assessments; in addition, it can be applied to help monitor and evaluate food and nutrition security.

## Introduction

Wheat accounts for 21% of global food crops, with a production of 766 million tons in 2019 (FAO, 2020), and it is one of the most important foods for human survival. The viscoelastic properties of dough made with wheat allow it to be formed into a variety of baked goods, which require the highest possible flour quality. Grain protein concentration (GPC) is the main descriptor for indicating flour quality; it affects the formation of gluten in bread production and the technological properties in baked products and determines the monetary value of wheat grain (Asseng et al., 2019). However, GPC alone may not be a suitable parameter for evaluating flour quality, which is a complex parameter and needs to be determined by combining GPC and composition characteristics (Chaudhary et al., 2016).

Grain proteins are polymorphic and based on their solubility properties, can be divided into various components: albumins, globulins, gliadins, and glutenins. Albumin and globulin have high nutritional value as well as structural and metabolic functions (Shewry et al., 1995). Gliadins and glutenins are gluten proteins, and their composition is decisive for flour quality, accounting for roughly 80% of the protein in wheat flour (Shewry and Halford, 2002). Specifically, the viscosity and ductility of dough are highly influenced by gliadins, while the strength and elasticity of the dough are mainly influenced by gliadins (Wieser, 2007). Increasing the nitrogen (N) content and changing the N distribution would increase the GPC and improve the composition by changing N partitioning in the grain proteins, thus enhancing flour quality and helping to ensure food and nutrition security (Zörb et al., 2010; Xue et al., 2016).

**FIGURE 1:**
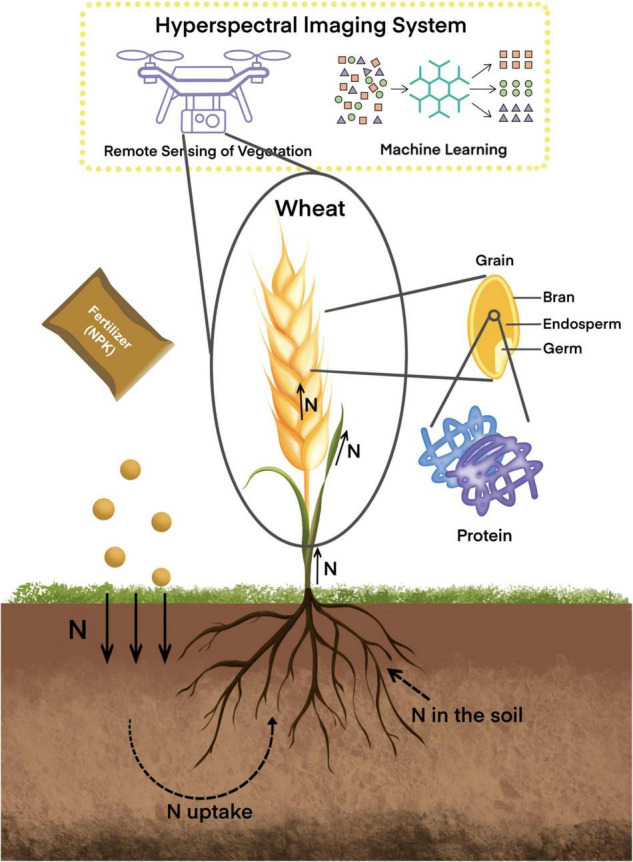
It is a review of hyperspectral imaging systems for evaluating wheat grain protein. Hyperspectral imaging systems, a combination of hyperspectral remote sensing and machine learning, have significant advantages in evaluating wheat grain proteins. Hyperspectral remote sensing can capture information reflecting nitrogen (N) status in wheat plants in real-time and non-destructively. Meanwhile, machine learning can effectively simulate the non-linear relationship between nitrogen and spectral data of wheat. Hyperspectral imaging systems are now widely used to predict wheat grain protein content (GPC), and crop models can complement the analysis of eco-physiological mechanisms in the prediction process.

N is absorbed throughout the growing season as an essential nutrient as illustrated in Figure 1 (Hawkesford, 2017). It is absorbed by seedling roots to supply the seed, as the seed’s reserves are rapidly exhausted. It continues to be absorbed during growth, driving the establishment of the canopy before anthesis. N uptake is highest around jointing and does not continue to rise after heading. Changes in N uptake, accumulation, and further partitioning within the plant lead to variations in the final GPC and its components (Hirel et al., 2007; Gaju et al., 2011). Differences in N uptake between the pre- and post-anthesis periods may affect N partitioning in wheat plants (Bogard et al., 2010) and the N content in the grains, as this mainly comes from two different sources: N stored in vegetative organs during the pre-flowering stage and N absorbed from the soil after flowering. Grain N is mainly remobilized from senescing canopy tissues and from the soil through the roots. Furthermore, N uptake efficiency is mainly related to the ability of the plant to maintain root activity and/or the plant’s ability to regulate N uptake during the grain-filling period (Foulkes et al., 2009; Hawkesford, 2014). The process of post-anthesis N remobilization to the grain affects the final GPC and composition during grain filling (Barraclough et al., 2010; Gaju et al., 2014), with the leaves and stems being the most valuable sources of N to the grain (Gaju et al., 2014). Canopy reflectance has been widely reported to be a good indicator of the N status in wheat plants because it is related to chlorophyll (Chl) *a* and *b* content (Wang et al., 2004; Zhao et al., 2005; Reyniers and Vrindts, 2006). Therefore, studying the eco-physiological characteristics of wheat canopy N during the growth period can provide a way to obtain field information in real time for agricultural production and can inform the breeding of high-yielding and good-quality wheat.

Numerous studies have already shown the potential of hyperspectral imaging techniques to estimate the quality of wheat grain (Figure 1). Traditional methods of assessing wheat N status requires destruction of the plant for chemical analysis. Although this method is more accurate, it is also time-consuming and laborious. To monitor the N status in wheat plants in real time and non-invasively, hyperspectral remote sensing has gradually been applied in recent years. This new method also reflects the spatial and temporal variation of N during the growing season using appropriate algorithms, which allows for recommendations before mid-season fertilization (Feng et al., 2008; Saberioon et al., 2014; Moharana and Dutta, 2016; Raya-Sereno et al., 2022). It is helpful for the early diagnosis of N stress, to inform remedial measures to ameliorate the stress. Moreover, it is of great significance to study the effect of environmental conditions on wheat grain quality.

Spectral data obtained from hyperspectral remote sensing instruments have a non-linear relationship with N in wheat plants. Hyperspectral vegetation indices (VIs) and machine learning are gradually being applied for the assessment of N status in wheat plants (Ranjan et al., 2012; Camps-Valls et al., 2018). The objectives of this study were (1) to investigate the existing wheat N-related hyperspectral VIs, which were inferred by hyperspectral inversion, with the aim of providing a reference method for selecting VIs in agricultural fields; (2) to summarize machine learning algorithms that can analyze field-derived hyperspectral data for wheat N status assessments; and (3) to explore the main challenges and future directions for the continued development of predicting wheat GPC and composition.

## Hyperspectral Techniques and Canopy Spectral Vegetation Indices of Nitrogen Status in Wheat Plants

Hyperspectral analysis can be used as a high-throughput phenotyping tool for assessing the N status in wheat plants during the growing season (Figure 1). Optical remote sensing techniques can measure eco-physiological traits in a high-throughput manner in field trials. Remote sensing of vegetation is mainly achieved by passive sensors that acquire electromagnetic wave reflection information from the canopy. It has been established that the reflectance of a wheat canopy in terms of its electromagnetic spectrum (spectral reflectance or emission characteristics of the canopy) is determined by the morphological and chemical characteristics of the leaf surface (Zhang and Kovacs, 2012). This data typically ranges from 350 to 2,500 nm, with unique reflectance value profiles in the visible (400–700 nm), near-infrared (700–1,200 nm), and short-wave infrared regions (1,200–2,500 nm), which are generally used to infer wheat plant growth characteristics (Serbin et al., 2015; Heckmann et al., 2017; Yendrek et al., 2017). Remotely-sensed data that indicate the growth, vigor, and N dynamics of a wheat canopy can provide necessary information for estimating grain quality and beneficial insights for agricultural production (Xue and Su, 2017).

In recent years, progress has been made in studying canopy spectral reflectance VIs in wheat plants with respect to N. When sunlight hits a wheat plant, most of the irradiance is consumed by water transpiration, and a small portion is used for CO_2_ assimilation. Part of the light energy is absorbed by the canopy and a part is reflected to space. The reflected light in the visible region of the electromagnetic spectrum is influenced by the chlorophyll pigment content in the wheat canopy, which in turn is related to the concentration of leaf N (Thomas and Gausman, 1977; Wessman, 1990). Blue (450 nm) and red (670 nm) wavelengths are two major absorption bands, due to the uptake of Chl *a* and *b*, the two main leaf pigments in wheat, which account for approximately 65% of the total pigment concentration in wheat plants. Therefore, it is possible to rapidly estimate the N status of wheat plants by remotely sensing the canopy Chl content.

The VIs, which are derived from wheat canopy hyperspectral reflectance, are used to describe vegetation characteristics that depend on the environment. The list of indices in Table 1 summarizes 20 VIs that quickly provide information on the N status of the entire wheat plant under field conditions (Gamon et al., 1997; le Maire et al., 2004; Herrmann et al., 2010). VIs for predicting Chl contents are usually based on (i) reflectance values far from the pigment absorption maxima and (ii) the selection of wavelengths close to the absorption bands. Another exciting region of the spectral area is the region between the strong red light absorption by Chl (680 nm) and the highly reflective near-infrared wavelengths (780 nm), a region of the spectrum known as the “red edge;” several red edge indices have been described (Vogelmann et al., 1993; Filella and Penuelas, 1994; Barnes et al., 2000). In wheat plants, Chl reduction caused by N deficiency leads to increased reflectance in the visible range (400–700 nm), changing the spectral signature, which reduces symptom-specific spectral characteristics (Osborne et al., 2002). Conducting experiments under diverse ecological conditions is helpful for validating other known VIs and for developing more broadly applicable monitoring models to indirectly assess the eco-physiological traits associated with N stress (El-Hendawy S. E. et al., 2019). Rodriguez et al. (2006) tested several developed VIs for estimating the level of canopy N nutrition in wheat plants under different environmental conditions and they derived a simple canopy reflectance index entirely independent of environmental factors. A N stress-free VI was developed that adjusts shoot %N according to the plant’s biomass and area; thus, it considers the environmental conditions affecting wheat growth.

**TABLE 1:** The 27 selected vegetation indices (VIs) that have been applied to wheat under field conditions were reviewed in the study, together with their number of spectral bands, band-specific formulations, and associated principal reference, including 17 two-band VIs and 10 three-band VIs.

Number of Bands	Vegetation Indices	Full Name	Formulation	References
Two-bands	CI_red edge_	Red Edge Model	(R_800_/R_700_)− 1	Jin et al., 2013
	EVI_800,660_	Enhanced Vegetation Index	2.56(R_800_−R_660_)/(1+R_800_+ 2.4R_660_)	Jin et al., 2013
	GI	Green Index	R_554_/R_677_	Duan et al., 2019
	NDSI_860,720_	Normalized Difference Spectral Indices based on the original spectrum	(R_860_-R_720_)/(R_860_+R_720_)	Yao et al., 2010
	NDSI_FD860,FD720_	Normalized Difference Spectral Indices based on the First Derivative spectrum	(FD_860_-FD_720_)/(FD_860_+FD_720_)	Yao et al., 2010
	NDVI	Normalized Differenced Vegetation Index	(R_790_-R_660_)/(R_790_+R_660_)	Hansen and Schjoerring, 2003; Hassan et al., 2019
	NDWI	Normalized Difference Water Index	(R_860_-R_1240_)/(R_860_+R_1240_)	Tuvdendorj et al., 2019
	NWI_970,990_	Normalized Water Index (R_970_, R_990_)	(R_970_-R_900_)/(R_970_+R_900_)	Babar et al., 2006
	NWI_970,850_	Normalized Water Index (R_970_, R_850_)	(R_970_-R_850_)/(R_970_+R_850_)	Babar et al., 2006
	NPCI	Normalized Pigments Chlorophyll Ratio Index	(R_680_-R_430_)/(R_680_+R_430_)	Tan C.-W. et al., 2018
	ONLI	Optimized Non-Linear Index	$1.5\left(0.6R_{798}^{2}-R_{728}\right)/\left(0.6R_{798}^{2}+R_{728}+0.05\right)$	Feng et al., 2019
	OSAVI	Optimized Soil-Adjusted Vegetation Index	1.16(R_800_-R_670_)/(R_800_ + R_670_ + 0.16)	Jin et al., 2013
	PRI	Photochemical Reflectance Index	(R_531_−R_570_)/(R_531_ + R_570_)	Robles-Zazueta et al., 2021
	RSI_990,720_	Ratio Spectral Indices based on the original spectrum	R_990_/R_720_	Yao et al., 2010
	RSI_FD725,FD516_	Ratio Spectral Indices based on the First Derivative spectrum	FD_990_/FD_720_	Yao et al., 2010
	RVI_870,660_	Ratio Vegetation Index	R_870_/R_660_	Zhu et al., 2008
	RVI_810,660_	Ratio Vegetation Index	R_810_/R_660_	Zhu et al., 2008
Three-band	EVI	Enhanced Vegetation Index	2.5[(R_900_−R_680_)/(R_900_ + 6R_680_−7.5R_475_ + 1)]	Wang et al., 2012; Robles-Zazueta et al., 2021
	MCARI_705,750_	Modified Chlorophyll Absorption Ratio Index calculated with reflectance from 705 to 750 nm	[(R_750_-R_705_)− 0.2(R_750_-R_550_)](R_750_/R_705_)	Wu et al., 2008
	MCARI2	Modified Chlorophyll Absorption Ratio Index Improved	$1.5\left[2.5\left(R_{803}-R_{671}\right)-1.3\left(R_{803}-R_{549}\right)\right]/\sqrt{\left(2R_{803}+1\right)2-\left(6R_{803}-5\sqrt{R_{671}}\right)-0.5}$	Haboudane et al., 2004
	mNDVI	Modified Normalized Differenced Vegetation Index	(R_924_−R_703_ + 2R_423_)/(R_924_-R_703_- 2R_423_)	Wang et al., 2012
	MTVI2	Modified Triangular Vegetation Index Improved	$1.5\left[2.5\left(R_{800}-R_{550}\right)-2.5\left(R_{670}-R_{550}\right)\right]/\sqrt{\left(2R_{800}+1\right)2-\left(6R_{800}-5\sqrt{R_{670}}\right)-0.5}$	Li et al., 2019
	MTCI	Medium Terrestrial Chlorophyll Index	(R_750_-R_710_)/(R_710_+R_680_)	Tan C.-W. et al., 2018
	SIPI-1	Structure Insensitive Pigment Index-1	(R_800_−R_445_)/(R_800_−R_680_)	Robles-Zazueta et al., 2021
	SIPI-2	Structure Insensitive Pigment Index-2	(R_800_−R_435_)/(R_415_−R_435_)	Robles-Zazueta et al., 2021
	TCARI_670,700_	Transformed Chlorophyll Absorption Reflectance Index	3[(R_700_−R_670_)−0.2(R_700_−R_550_)(R_700_/R_670_)]	Wang et al., 2012; Wu et al., 2021
	TCARI_705,750_	Transformed Chlorophyll Absorption Reflectance Index calculated with reflectance from 705 to 750 nm	3[(R_750_−R_705_)−0.2(R_750_−R_550_)(R_750_/R_705_)]	Wu et al., 2008

*R_λ_ is the spectral reflectance of random wavelengths (λ); FD_λ_ is the corresponding derivative spectrum.*

Two-band VIs are increasingly used for N estimation (Table 1). VIs are calculated from canopy reflectance values for specific visible and near-infrared wavelengths (Frels et al., 2018). These indices can estimate changes in canopy Chl content and thus indicate the N status of wheat plants (Gutierrez et al., 2004). Hansen and Schjoerring (2003) used two-band combinations of the normalized vegetation index (λ1 − λ2)/(λ1 + λ2) to predict N-related crop variables at different growth stages during the winter wheat growing season in a field experiment. Zhu et al. (2008) identified common spectral bands and VIs to characterize the N status of wheat leaves and analyze the quantitative relationship between leaf N status and canopy reflectance. In their study, ratio VIs (RVIs) (R_870_, R_660_) and RVIs (R_810_, R_660_) showed the highest correlation with leaf N status compared to other specific RVIs, differential VIs (DVIs), and normalized VIs (NDVIs) in the 16 bands from the MSR16 radiometer. Yao et al. (2010) conducted real-time monitoring of wheat canopy hyperspectral reflectance and leaf N status under different treatments in a field experiment. They found that the sensitive spectral bands of the leaf N status concentrated in the visible and near-infrared regions, and that NDSIs (R_860_, R_720_), RSIs (R_990_, R_720_), NDSIs (FD_736_, FD_526_), and RSIs (FD_725_, FD_516_) were the best VIs for estimating the N status in wheat plants. However, the values of VIs calculated by combining two bands were found to be closely related to the number of leaves in the canopy. A dense canopy can quickly saturate the two bands, making it less sensitive to the plant’s high eco-physiological content (Gitelson, 2004). Therefore, three-band VIs were developed to solve the problem of canopy saturation that can occur in two-band VIs (Wang et al., 2012).

Selecting the optimal central bands for three-band VIs requires a comprehensive analysis of two-band VIs based on hyperspectral information (Hansen and Schjoerring, 2003; Zhu et al., 2008; Yao et al., 2010). Selecting the central band of the three-band VIs is a bandwidth issue, and is controversial in wheat plant growth monitoring. Broge and Mortensen (2002) predicted eco-physiological traits in wheat at three newton levels in the field by comparing the predictive power of broadband-based VIs with narrowband-based VIs. They concluded that narrowband-based VIs were more sensitive to changes in wheat plants during growth and more effective at minimizing noise and saturation in eco-physiological trait estimation. In contrast, Wang et al. (2012) conducted field experiments with different N levels, moisture conditions, and wheat varieties, and concluded that broadband-based VIs are more realistic. Therefore, they constructed a new three-band VI by combining narrowband-based VIs and broadband-based VIs to reduce the saturation of broadband-based two-band VIs on the basis of reality (Table 1). Based on this, a reliable and stable linear monitoring model for leaf N concentration was established, which provides a good index and an accurate estimation model for monitoring the N status in wheat plants using a three-band VI (Wang et al., 2012).

There is little agreement in previous studies on which VIs are most suitable for determining the N status in wheat plants. Main et al. (2011) investigated 73 VIs and ranked them according to their relationship with total Chl content, which is related to N. They found that indices using the red-edge region (680–730 nm) were better predictors of the N status of wheat canopy (via canopy Chl content), with a better linear relationship and lower saturation than other VIs. Frels et al. (2018) used 299 wheat genotypes in a 2-year trial near Ithaca, NE, United States, to compare the correlation between 28 VIs inferred from canopy spectral reflectance during the grain filling stage and N-related traits in wheat plants. They concluded that it is more accurate to use VIs to estimate N traits in wheat plants during the early grain filling stage, as more traits were associated with the Maccioni index. This index captures many components of the N use efficiency. Incorporating it into the existing selection programs could yield more N-related indicators (Frels et al., 2018).

## Dissection of Hyperspectral Reflectance to Estimate Nitrogen Status in Wheat Plants Based on Machine Learning Algorithms

Machine learning is an effective method for solving complex problems such as multicollinearity and overfitting in multiple linear models (MLMs) to estimate the N status in wheat plants (Figure 1). With the development of hyperspectral imaging systems, the amount of computation required has gradually increased. Moreover, the massive data features tend to cause overfitting and affect estimations produced by MLM. Selecting a suitable estimation method can reduce the dimensionality of the raw data, screen out necessary information from the data, significantly improve the validity of the data, and is an important aspect of improving the accuracy of N status estimation in wheat plants (Li D. et al., 2020). Most studies have used MLM to quantitatively assess the relationship between spectral indices and N status (Babar et al., 2006; Pavuluri et al., 2015). However, when many characteristic dimensions are used, the correlations between VIs and leaf N status are generally low, and the models are prone to multicollinearity and overfitting, which reduces the accuracy of the estimated N status (El-Hendawy S. et al., 2019). To address these issues, machine learning methods can reduce the wide range of co-linear variables and non-correlated factors, and reduce the impact of background effects on model precision (Singh et al., 2021).

Machine learning techniques are advantageous for estimating agricultural indices from hyperspectral remote sensing data (Zhang et al., 2021). Wheat canopy reflectance is a function of the wheat leaf’s optical properties, wheat canopy structure, soil background, atmospheric conditions, observation geometry, and solar zenith angle (Boegh et al., 2002; Baret et al., 2007; Homolová et al., 2013). The relationship between canopy reflectance and wheat N status is controlled by many influencing factors. Thus, finding the response mechanism of canopy hyperspectral reflectance to the wheat N status is often complicated and nonlinear in character (Fu et al., 2021). Most machine learning algorithms are often considered “black boxes” because they provide no information about how they work. Therefore, machine learning can be used to explore the complex non-linear relationships between spectral features and the N status in wheat plants without a clear understanding of the original data distribution (Singh et al., 2021). This not only provides a multi-faceted and flexible direction for data analysis, but also a wider scope for experts to apply their theoretical knowledge to explain the principles in conjunction with algorithms. Previous spectral indices have depended on a few available spectral bands and, therefore, do not use all the information conveyed by the spectral trace. In contrast, machine learning techniques not only use inverse VIs but also all the spectral information, demonstrating the potential to analyze hyperspectral reflectance data with a large number of bands and to evaluate additional features. This provides more information for high-precision prediction modeling of the N status in wheat plants (Chlingaryan et al., 2018). Recently, the advantages of machine learning over VI-based approaches have been highlighted. Specifically, machine learning has excelled in modeling the complex mechanisms of canopy-scale spectral features in response to the N status in wheat plants, without the need to know the underlying data distribution (Thorp et al., 2017; Miphokasap and Wannasiri, 2018; Tan K. et al., 2018). With the proliferation of spaceborne, airborne, and unmanned aerial vehicle (UAV) imaging spectrometers, many types of hyperspectral data are now available and has ushered in the era of “big data” in the field of remote sensing. This requires machine learning algorithms to mine available information to more effectively monitor the N status in wheat plants in real time and to predict their GPC and composition (Fu et al., 2021).

Machine learning involves various types of learning techniques. Partial least squares regression (PLSR) is a commonly used technique for estimating eco-physiological traits in wheat from hyperspectral data (Fu et al., 2014). Hansen and Schjoerring (2003) calculated NDVIs for all possible combinations of wavelengths in the range of 438–884 nm. They found that linear regression and PLSR can be used to estimate Chl and N concentrations in wheat plants. Pimstein et al. (2007) also used PLSR to estimate the N content of wheat leaves from spectral (350–2,500 nm) data collected in the field. The authors concluded that the appropriate wavelength and application of derivatives to the raw spectra could improve the predictive quality of the estimated model. Li et al. (2014) also estimated canopy N content in wheat plants using optimized hyperspectral VIs combined with PLSR. Mahesh et al. (2015) used PLSR to predict wheat GPC based on hyperspectral reflectance. In conclusion, PLSR has been applied as a method capable of analyzing a large amount of noise-laden co-linear data to monitor N content in wheat plants and predict wheat GPC in agricultural fields. Its accuracy improves with an increase in the number of relevant variables and observations (Berger et al., 2020).

In addition to PLSR, kernel-based regression methods are becoming more popular in wheat N status assessment using hyperspectral data. Machine learning algorithms require a few statistical assumptions to be applied to the data to develop linear and nonlinear models. Among them, kernel-based regression methods [e.g., support vector regression (SVR) and Gaussian process regression (GPR)] use structural risk minimization. Therefore, with a limited training set, these methods are considered to have a better generalization ability than artificial neural networks (ANNs) (Fu et al., 2020). Li et al. (2016) compared four chemometric techniques used to estimate N status in winter wheat plants using spectral features. In their study, the predictive power and the impact of sample size were assessed. They proposed that SVR is more suitable than back-propagation neural networks (BPNN) for estimating winter wheat N concentrations when the sample size is insufficient. Different kernel functions differ in their ability to embed geometric structures in the training samples. To combine the advantages of different kernel functions, Wang et al. (2017) constructed a multiple-kernel SVR (MK-SVR) consisting of a radial basis function (RBF) kernel and polynomial kernel for N status estimation in wheat plants. They found that the MK-SVR outperformed multiple linear regression (MLR), partial least squares (PLS), ANNs, and single kernel SVR (SK-SVR) models, introducing a new method for non-destructive and rapid monitoring of the N status in wheat plants based on hyperspectral data.

Physical and hybrid methods have also been applied to wheat N-inversion. Their application is based on the high degree of correlation between leaf Chl and the N status in wheat plants, and their effectiveness depends on the radiative transfer models (RTM) used, the inversion technique applied, and the quality of the data measured (Danner et al., 2021). PROSAIL is a widely used radiative transfer model for estimating the N status in wheat because it provides a good compromise between model realism and inversion possibilities. Yang et al. (2015) developed an N-PROSPECT model to estimate the N status of the winter wheat canopy by replacing the Chl uptake coefficient with the N uptake coefficient in the original PROSPECT model. Coupling of the N-PROSPECT model and the SAIL model (N-PROSAIL) has been used to estimate canopy N density in wheat plants, and the model has been shown to have a high potential for establishing the N status in wheat plants (Li et al., 2018b,^2019^). However, the ability of PROSAIL and N-PROSAIL models to characterize a non-homogenous canopy structure before complete canopy closure is poor, resulting in less accurate estimates of the N status in wheat plants (Botha et al., 2010).

## Deficiencies and Prospects of the Hyperspectral Imaging System for Estimating the Nitrogen Status in Wheat Plants

The use of the hyperspectral imaging system often allows for immediate and punctuated estimates of the wheat N status over wide regions. However, there are intrinsic inevitable shortcomings in the empirical methods. Hyperspectral VIs are widely used in crop N estimation due to its simple, timely and efficient computation. Further, machine learning techniques are expected to be more suitable for simulating wheat N status from such data due to their ability to deal with nonlinear problems. Many combinations of hyperspectral VIs and machine learning algorithms have yielded improved N status estimates for wheat plants, and there have been studies modeling the relationship between VIs and GPC based on machine learning algorithms. For example, Tan et al. (2020) analyzed 14 VIs by using PLSR and found that 12 of them were closely related to wheat GPC (model prediction accuracy > 90%). Nevertheless, PLSR was not compared with other machine learning methods in the study. Although PLSR is superior to linear regression and principal component analysis model, decision tree, random forest, artificial neural networks and other methods may have better simulation results for wheat GPC. Furthermore, the hyperspectral imaging system still needs improvement. First, the results of models calibrated with hyperspectral data are significantly influenced by seasonal characteristics, cropping environments and experimental design, which is characterized by poor spatio-temporal heterogeneity. Therefore, applying a model from one specific environment or growing season to any other environment or growing seasons may result in poor prediction results of wheat grain nutrition (Colaço and Bramley, 2018, 2019). Second, the spectral features identified by the available feature mining techniques involve statistical models and largely depend on a dataset without any inherent relationships between GPC, the environment, and management, with unclear physical meaning and poor explanation of mechanism (Li Z. et al., 2020; Figure 1). Some researchers have argued that as (i) wheat growth is a dynamic process and (ii) wheat canopy N status changes dynamically; a better understanding of the physical processes underlying the hyperspectral response to wheat canopy N status is required. This would enable the development of robust assessment models with causal relationships that complement the eco-physiologically hyperspectral imaging systems (Li et al., 2018a; Fu et al., 2021).

The combination of crop models and remote sensing data is emerging as a promising approach for monitoring wheat growth and grain protein accumulation. Crop models can simulate the dynamic biological processes of wheat growth based on the quantitative relationships between wheat growth and environmental conditions, including weather, soil conditions, wheat genotype information, and field management (Jin et al., 2018). Crop models combined with hyperspectral data can provide time continuity for wheat quality prediction systems. This can enable the effective development of crops based on the environment and stress conditions (water or N), improve the temporal and spatial expansion ability of wheat quality prediction, enhance the agronomic and mechanical rationality of prediction, and can be used for scenario testing and strategic (long-term) decisions (Li Z. et al., 2020). In previous studies, the transfer of N to the grain has been simulated at various levels of complexity. Both simpler harvest indexing approaches [e.g., SIRIUS (Jamieson et al., 1998) and STATISTICS (Brisson et al., 1998)] and more sophisticated source-aggregation models [e.g., SIRIUS (Jamieson and Semenov, 2000)] illustrate this evolution. The CERES-Wheat grain filling program applied independent controls for dry matter and N accumulation in the grain, dividing the grain filling process into stages. However, variation in N accumulation due to genetic variance was not considered in this procedure (Ritchie et al., 1998). APSIM-Nwheat used the same grain protein program as CERES-Wheat and applied the model to study the effect of seasonal temperature and rainfall interactions on grain N concentrations (Asseng and Milroy, 2006). Meanwhile, other studies have proposed a framework to model the mechanisms of N uptake and partitioning in wheat plants, thus advancing toward more accurate modeling of N dynamics (Jamieson and Semenov, 2000). In recent years, crop models have been used to assess the impact of climate change on wheat GPC, however, uncertainty varies with location (Asseng et al., 2019).

In addition, the SiriusQuality model has been developed to consider the assignment of structural and storage proteins in wheat grain. Within the routine, the model divides N into structural/metabolic proteins and major storage proteins, and it provides predictions of the protein compositions, which are the alcoholic and glutenin fractions (Martre et al., 2003, 2006). The SiriusQuality model also assumes that the partitioning of N between the storage protein compositions, alcoholic, and gluten remains constant during the grain filling stage. It also assumes that the interactions between the genotype and environment alters the total grain N through source limitation rather than through partitioning of N between different protein compositions (Martre et al., 2006). This provides proof of concept that crop models can be extended to explain protein composition. In general, existing crop models can correctly simulate GPC in the absence of stress treatments; however, the performance of models under extreme temperature conditions still needs to be improved (Osman et al., 2020). In addition, some important model input parameters are difficult or impossible to obtain, and the information provided to crop models on wheat growth is limited to scattered points (Fu et al., 2021).

## Conclusion

An accurate assessment of the in-plant N status of wheat is essential for predicting GPC and composition, in addition to ensuring food and nutritional safety. We are at the beginning of a promising path toward using hyperspectral imaging systems to predict GPC and composition in wheat plants. On the one hand, as a high-throughput phenotypic tool, hyperspectral remote sensing has the potential to complement or even replace types of field measurements for some wheat N-related traits in the growing season. On the other hand, the combination of hyperspectral VIs and machine learning algorithms is a powerful tool for estimating agricultural indices from hyperspectral remote sensing data. However, VIs and the spectral features identified by machine learning algorithms in hyperspectral imaging systems depend heavily on the input dataset without any intrinsic relationship between GPC, the environment, and field management. Based on our review, we suggest that these issues can be understood in conjunction with crop models. Crop models consider the environmental effects of wheat growth. These methods strive to uncover the underlying mechanisms of wheat growth when using hyperspectral data to predict wheat GPC and composition. Insight into the limitations of these methods will help us select the appropriate method for monitoring the N status in wheat plants and contribute to further developing methods for wheat grain quality studies.

## Author Contributions

YH: conceptualization, funding acquisition, and writing-review and editing. JM and YH: formal analysis. JM, BZ, and YH: writing the manuscript, read, and agreed to the submitted version.

## Conflict of Interest

The authors declare that the research was conducted in the absence of any commercial or financial relationships that could be construed as a potential conflict of interest.

## Publisher’s Note

All claims expressed in this article are solely those of the authors and do not necessarily represent those of their affiliated organizations, or those of the publisher, the editors and the reviewers. Any product that may be evaluated in this article, or claim that may be made by its manufacturer, is not guaranteed or endorsed by the publisher.
